# Pediatric chemical burn with paint remover: A case report

**DOI:** 10.1097/MD.0000000000045256

**Published:** 2025-11-07

**Authors:** Ruiqun Zhang, Ying Dong, Jian Zhang, Yu Liu, Hailiang Zuo

**Affiliations:** a Department of Burn Surgery, Tianjin Children’s Hospital, Tianjin University, Tianjin, China.

**Keywords:** acute kidney injury, acute kidney injury, chemical burn, paint remover

## Abstract

**Introduction:**

Chemical burns occur when the human body is exposed to certain chemicals, causing immediate injury, continued invasion, or absorption, leading to progressive local damage or systemic poisoning. Despite the obvious presence of easily accessible toxic products in daily life, the incidence of chemical burns in children is increasing.

**Case presentation:**

A 4-year-old girl sustained extensive chemical burns and subsequent systemic toxicity after exposure to paint remover. This report summarizes the initial investigation, management plan, and treatment outcomes.

**Conclusions:**

Education and awareness are the most effective preventive measures against chemical burns.

## 1. Introduction

Chemical burns occur when the skin, mucosa, or other tissues are exposed to chemical substances, resulting in pathological damage such as degeneration and necrosis. Some chemicals can be absorbed through the skin, mucosa, or respiratory tract, causing systemic poisoning symptoms. Most chemical burns in children are commonly caused by household products, while cases involving industrial paint remover are less common. The composition of paint remover varies by manufacturer and type; however, it commonly contains dichloromethane (CH_2_Cl_2_ (40%)), complex organic acids (15%), and other auxiliaries (45%), as indicated in the material safety data sheet (TDT230312010CN).^[1]^ Dichloromethane is widely used due to its solubility and stripping properties. Four cases of chemical burns caused by paint removers have been reported, involving eye injury,^[2]^ diffuse pulmonary damage,^[3]^ reduced female visits,^[4]^ and acute hemorrhagic cystitis.^[5]^ In this report, we present the case of a 4-year-old girl who sustained extensive chemical burns from paint remover, leading to acute kidney injury (AKI).

## 2. Case presentation

A 4-year-old girl presented to our hospital with extensive chemical burns 2 hours after the injury. Her father, an employee at a paint factory, was driving a minivan when paint remover was accidentally spilled, causing her to slip and fall into the chemical.

**Figure 1.**
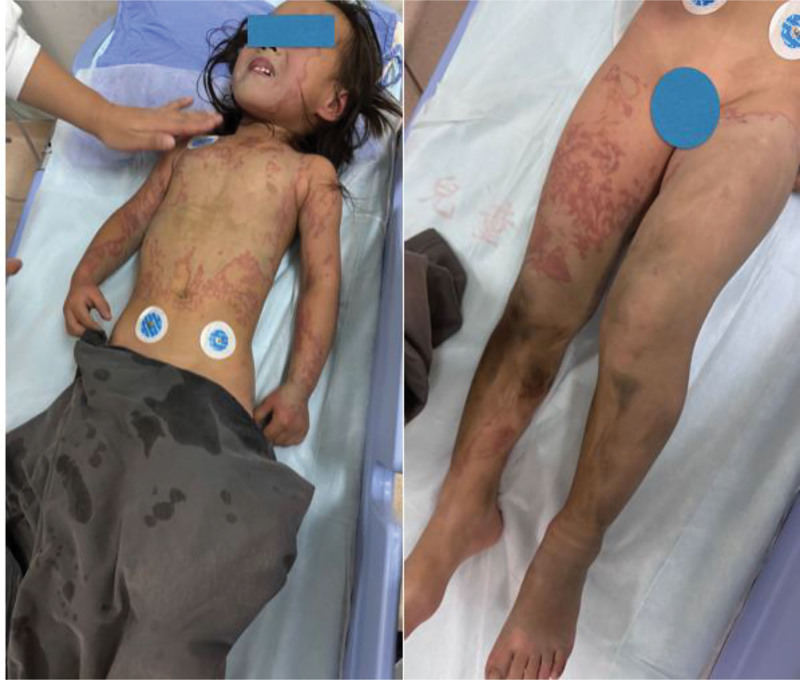
Clinical images of the patient on admission.

On physical examination, the patient exhibited tachycardia, reduced urine output, hematuria, and bilateral corneal injuries. Deep partial- to full-thickness chemical burns were present on the face, chest, and abdomen, as well as bilateral upper and lower extremities, covering approximately 40% of the total body surface area. The wounds appeared as stained white–brown eschars with well-defined borders and no blistering (Fig. 1). The patient had no significant medical, surgical, or social history.

Immediate treatment included copious irrigation with normal saline, with a particular focus on ocular irrigation, followed by aggressive fluid resuscitation and sodium bicarbonate administration to alkalinize the urine, aiming to protect and restore organ function. An ophthalmologist evaluated the patient. Topical steroids and antibiotics were applied to the eyes to reduce inflammatory damage.

Following fluid support, the patient’s urine output increased from approximately 0 mL/kg/h in the beginning to 1.55 mL/kg/h. However, 9 hours after admission, the patient developed oliguria again (0.48 mL/kg/h), accompanied by an elevated creatinine level (2.8 times the reference value of 60 μmol/L), leading to a diagnosis of AKI.

On day 1 at the hospital (post-burn day 1), the patient was transferred to the pediatric intensive care unit for continuous renal replacement therapy (CRRT) and further resuscitation. Pain management and sedation were provided, along with treatment of preserved eschar on the burn surface. The patient was regularly monitored by dietitians. CRRT was administered for 7 days until the patient regained voluntary urination function. However, her creatinine level remained elevated due to a high risk of sepsis and symptoms of a lower respiratory tract infection.

Though multiple blood and sputum cultures were performed, empirical therapy with meropenem and linezolid was initiated.

**Figure 2.**
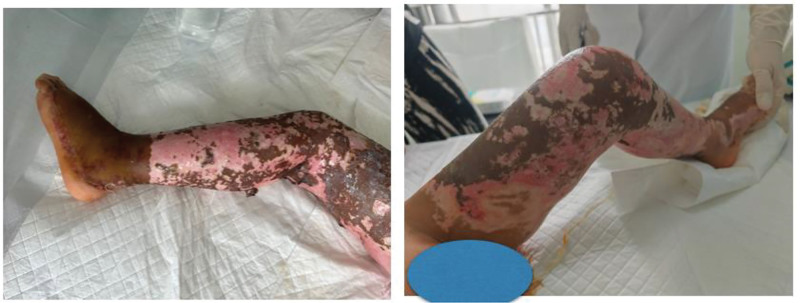
Clinical image of the wound surface eschar that started dissolving on day 10 after admission to the hospital.

On day 10 at the hospital, the patient developed a persistent fever as the necrotic eschars began to dissolve (Fig. 2). Her burns were debrided daily and dressed conservatively with silver sulfadiazine until her renal function recovered following AKI treatment on hospital day 22.

On day 23 at the hospital, the patient underwent operative debridement of devitalized tissue, followed by repeated debridement and autografting on day 32. A split-thickness skin graft obtained from her scalp was used to reconstruct her chest and abdomen, as well as bilateral upper and lower extremities, achieving 100% graft take.

**Figure 3.**
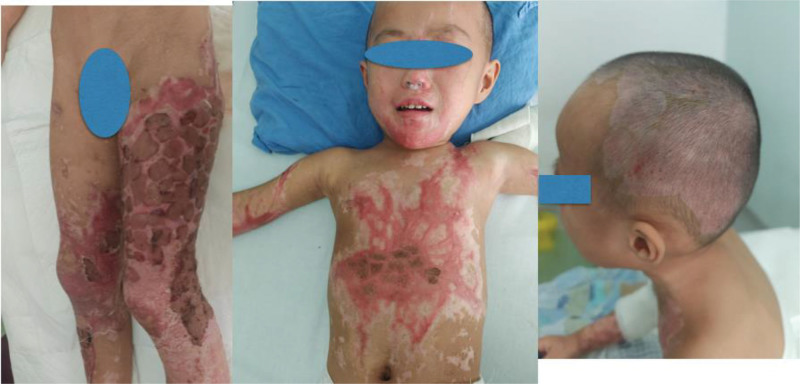
Clinical images of the patient at cured discharge.

On day 52, the patient underwent debridement and autografting for the remaining burns on the left buttock and left foot. By day 68, the grafted burn sites had nearly healed, and the patient was discharged with complete healing of the wound surface, superficial scar hyperplasia, and no current functional deficits (Fig. 3).

## 3. Discussion

The report emphasizes the importance of evaluating the management approach to gain insights from this case, aiming to improve future outcomes while ensuring optimal functional and aesthetic results.

The initial consideration was whether the timing of debridement was appropriate, given the AKI caused by chemical burns. Early debridement offers several advantages, such as the prevention of progressive necrosis, reduction of toxin absorption, and a decreased infectious burden. However, CRRT requires anticoagulation, which may lead to excessive intraoperative bleeding, increased transfusion requirements, and potential termination of the operation.^[6]^ Excessive bleeding is another pre-renal factor that can exacerbate renal dysfunction, thereby increasing the risk of permanent renal failure in children. Given these concerns, we chose the conservative approach until her renal function recovered, despite continuing tissue destruction.

Second, we focused on fluid resuscitation strategies specific to chemical burns and calculated the fluid requirement for the first 24 hours based on the formula adopted in the 1970 China Burn Medicine Seminar and the specific condition of the child, which indicated a need for 1.2 L of fluid.^[7]^ However, due to the presence of tachycardia, hematuria, and oliguria at admission, the patient required 3.4 L of fluid resuscitation in the first 24 hours, resulting in excessive fluid balance and the development of orthostatic edema on hospital day 1.

Determining whether aggressive fluid resuscitation contributes to interstitial edema, potentially exacerbating circulatory overload and deepening the wound, is challenging. Over-resuscitation can lead to complications such as compartment syndrome, pulmonary edema, acute respiratory distress syndrome,^[8]^ as well as an increased probability of fascial incision and skin grafting.^[9]^

Researchers have proposed the concept of permissive oliguria,^[10]^ setting a target urine output of 0.5 mL/kg/h as the for permissive hypovolemic fluid resuscitation in pediatric burns.^[11]^ Maintaining organ function while preventing over-resuscitation requires a delicate balance.

Finally, this case highlights the consequences of inadequate awareness regarding the toxic effects of this commonly used chemical. Furthermore, the initiation of continuous eye and skin decontamination with water is critical. We hope to emphasize that prevention is more important than treatment, especially in children. Additionally, education and awareness of the consequences of chemical burns are the best preventive measures.

## Acknowledgments

We would like to thank Editage (www.editage.cn) for English language editing.

## Author contributions

**Writing – original draft:** Ruiqun Zhang.

**Investigation:** Yu Liu.

**Project administration:** Jian Zhang.

**Supervision:** Hailiang Zuo.

**Writing – review & editing:** Ying Dong.
